# Transcriptomic Profile of Glioblastoma Cells Infected with Zika Virus: A Systematic Review and Pathway Analysis

**DOI:** 10.3390/v18020249

**Published:** 2026-02-15

**Authors:** Diego Menezes, Clarisse Rezende Reis, Izabela Mamede, Victor Emmanuel Viana Geddes, Renan Pedra de Souza, Renato Santana Aguiar

**Affiliations:** 1 Laboratory of Integrative Biology, Department of Genetics, Ecology and Evolution, ICB, UFMG, Belo Horizonte 31270-901, Brazil; 2 Laboratory of Genetics Biochemistry, Department of Biochemistry and Immunology, ICB, UFMG, Belo Horizonte 31270-901, Brazil; 3 D’OR Institute for Research and Education, Rio de Janeiro 22281-100, Brazil; 4 IDOR Pioneer Science Initiative, São Paulo 01401-002, Brazil; 5 René Rachou Institute, Oswaldo Cruz Foundation, Belo Horizonte 30190-002, Brazil

**Keywords:** oncolytic viruses, glioblastoma, Zika virus (ZIKV), transcriptome, pathway analysis

## Abstract

Glioblastoma (GBM) is an aggressive tumor with limited therapeutic options. Zika virus (ZIKV) has demonstrated activity against GBM; however, the cellular pathways behind this interaction remain unclear. We systematically reviewed open-access primary studies assessing differentially expressed genes (DEGs) in GBM models infected with wild-type or engineered ZIKV using transcriptomic approaches (inclusion criteria); reviews, restricted-access studies, commentaries, preprints, abstracts, and articles lacking data or not meeting these conditions were excluded (PROSPERO CRD420251077092). We performed a pathway analysis of reported DEGs. PubMed and Google Scholar were searched up to 5 March 2025; 139 records were identified and 5 met the eligibility criteria. Risk of bias was evaluated using an adapted ToxRTool for in vitro experiments and the SYRCLE RoB tool for in vivo models. Altogether, 4360 genes were reported as upregulated and 2072 as downregulated; 12 genes (*DNAJB9*, *SESN2*, *PMAIP1*, *PPP1R15A*, *KLF4*, *ATF3*, *IFNB1*, *IFNL1*, *ANKRD33B*, *ZC3HAV1*, *OASL*, and *CCL5*) were consistently upregulated, none were consistently downregulated. Pathway analysis of the studies providing complete DEG lists identified 23 commonly enriched pathways mostly related to interferon signaling. These findings may help guide future research in this field; nevertheless, methodological heterogeneity limits comparability, reinforcing the need for standardized protocols. Funding: ITpS, CNPq, and FAPEMIG.

## 1. Introduction

Glioblastoma (GBM) is a primary malignant glial tumor (glioma) that originates from the differentiation of astrocytes. It is classified as a grade IV tumor on the World Health Organization (WHO) malignancy scale [1], representing the most aggressive and the most common type of central nervous system (CNS) cancer. With an estimated 10,500 new diagnoses per year in the United States, GBM accounts for approximately 80% of all primary malignant CNS tumors [2]. The average age at diagnosis is 64 years and the median overall survival following diagnosis ranges from 12 to 15 months [3,4]. The combination of the tumor’s aggressiveness and the scarcity of effective therapeutic options has intensified the demand for targeted treatments, fostering a growing research line within the field of oncology that offers hope for patients who currently lack viable medical alternatives.

Among the promising therapeutic strategies to emerge over the past decade is immunotherapy, which seeks to harness the immune system to target and eliminate tumor cells [5]. In parallel, the field of oncolytic virotherapy has emerged as an even bolder approach. While immunotherapy focuses on modulating host immune responses against tumor cells, oncolytic virotherapy relies on the selective infection and replication of viruses within malignant cells, resulting in direct oncolysis. These oncolytic viruses (OVs), whether wild-type or genetically engineered, have been experimentally proven to selectively replicate within cancer cells, leading to their destruction without harming healthy cells in the host organism [6].

As of now, 13 clinical trials investigating OVs for GBM treatment have been registered, including models based on herpesviruses, adenoviruses, parvoviruses, polioviruses, and reoviruses [7]. More recently, scientific interest in Zika virus (ZIKV) has increased following the outbreaks of ZIKV-associated fever in the Americas [8]. Research on ZIKV as a potential OV against GBM reported its first positive results in 2017, when Zhu and collaborators demonstrated oncolytic activity in three GBM cell lines [9]. Since then, numerous studies have been published, aiming to investigate the molecular mechanisms underlying this effect, primarily through targeted molecular and mechanistic strategies [10,11,12,13]. Although these works have significantly contributed to the field, hypothesis-free exploratory approaches, such as global cellular pathways profiling via total RNA sequencing (RNA-seq), are widely advocated for emerging biological phenomena, as they provide an unbiased foundation for the development of new hypotheses. Despite increasing interest in transcriptomic profiling of ZIKV-infected GBM models, the literature remains difficult to synthesize due to the diversity of experimental systems and analytical strategies commonly employed in this research area. In this context, a systematic review that integrates the available evidence from multiple sources is warranted.

This study systematically reviews the available evidence on the transcriptomic landscape of ZIKV-infected GBM cells, emphasizing the key genes implicated in this process. In addition, we performed a pathway analysis using the differentially expressed genes (DEGs) reported in the included studies to integrate findings across different transcriptomic reports.

## 2. Materials and Methods

We registered a study protocol on the International Prospective Register of Systematic Reviews (PROSPERO: CRD420251077092). Preferred Reporting Items for Systematic reviews and Meta-analysis (PRISMA) was adopted as a guideline for reporting this systematic review [14]. Inclusion criteria were open-access primary studies that assessed DEGs in GBM models infected with ZIKV using a transcriptomic approach. Eligible studies can use either wild-type ZIKV or various ZIKV-based engineered models as the main intervention, as well as different GBM models as the experimental system. Exclusion criteria were reviews, restricted-access studies, commentaries, editorials, preprints, conference abstracts, and other articles lacking full data. Additionally, primary studies were excluded if they did not assess DEGs through a transcriptomic experimental design, did not use wild-type ZIKV or ZIKV-based engineered models as the main intervention, or did not employ a GBM model as the experimental system.

Study selection was conducted in three phases: database search (identification), title and abstract screening, and full-text assessment for eligibility. Identification was performed by searching on two databases: PubMed and Google Scholar. Cross-referencing was used to identify additional records and studies from the reference lists of the reviews retrieved in our search. We included all studies published up to 5 March 2025, using the search arguments listed in the Supplementary Material (Supplementary File S1). Two independent researchers conducted the screening and full-text eligibility assessment. A systematic review flowchart was prepared following PRISMA specifications (Figure S1). Only studies using human GBM models that provided the complete list of DEGs, either reported within the article or deposited in public databases, were selected for gene overlap assessment and pathway analysis; therefore, no additional methods to assess bias from missing results were necessary.

Data were manually extracted by a single reviewer (DM) without the use of standardized forms. Full-text articles were individually screened, and relevant information was systematically collected and entered into a structured outcome table, which was subsequently reviewed by all co-authors. The table included the following variables: Study, Virus strain/viral model, Multiplicity of Infection (MOI) or PFU/cell, RNA collection time (hpi), Species, Model, Method, Outcome, Mean sequencing depth (million reads/sample), Results (DEGs), and Observations (Table 1). In the Results (DEGs) section, genes were classified as upregulated or downregulated. For DEGs identified via Reverse Transcriptase quantitative Polymerase Chain Reaction (RT-qPCR), gene symbols were listed. For those obtained via RNA-seq, only the total number of genes in each category was reported, in order to preserve the table’s clarity and readability.

All DEGs were manually curated using the Multi-Symbol Checker provided by the HUGO Gene Nomenclature Committee (HGNC) [15] to standardize gene nomenclature. Gene identifiers from Ensembl [16] and NCBI [17] were mapped to approved HGNC gene symbols. Only approved symbols were retained, while unmatched entries, aliases, and deprecated symbols were excluded to avoid redundancy and to ensure consistency across studies. A final dataset was compiled, including all DEGs identified through RNA sequencing between infected and non-infected human GBM models, together with study authorship and the corresponding expression pattern (Table S1). From this dataset, overlapping genes among studies were assessed through Venn diagrams generated in R [18], with the support of the following packages: readxl [19], dplyr [20], tidyverse [21], VennDiagram [22], png [23], ggplotify [24], ggpubr [25], and grid [18].

The risk of bias was assessed by two independent researchers using one of two instruments, depending on the study design. In vitro studies were evaluated using the ToxRTool [26], a tool developed to assess the reliability of toxicological data, which classifies studies into Klimisch categories: reliable without restrictions (score 1), reliable with restrictions (score 2), and not reliable (score 3) [27]. The final score assigned to each study was included in the outcome table under the variable ToxRTool score. Since there are no standardized tools for evaluating bias in virotherapy-based in vitro studies to the present date, ToxRTool was adapted for the context of this review because its criteria for reliable toxicological data, including test substance characterization and detailed description of study design, are broadly applicable to assessing the methodological rigor of in vitro virotherapy studies; the adaptation criteria are detailed in the Supplementary Material (Supplementary File S2). In vivo studies were assessed using SYRCLE’s Risk of Bias Tool [28], an instrument based on the Cochrane Risk of Bias Tool, applicable to any type of intervention in preclinical animal studies.

To investigate pathway alterations across the articles, we performed gene set enrichment analysis using the fgsea [29] package in R. Publicly available RNA-seq differential expression (DE) results were retrieved and processed as follows: Count matrices were processed using the DESeq2 pipeline [30]. Gene names were standardized using bioMart [31]. Gene set enrichment was performed against the C5 Gene Ontology Biological Process (GO:BP) collection (version 2025.1) from MSigDB [32]. All tested genes per comparison were included in fgsea analysis and they were ranked by their log2FoldChanges. Pathways with adjusted *p*-values < 0.01 and |normalized enrichment score (NES)| > 1 were considered significantly enriched. The top 10 absolute NES pathways in each dataset were intersected for visualization in ggplot2 [33]; since some pathways were present in the top 10 of more than 1 dataset, this amounted to 23 pathways. No formal analyses to explore heterogeneity, sensitivity analyses, or certainty assessments were conducted, as the synthesis was descriptive, based on fixed thresholds, and focused on transcriptomic pathway data rather than quantitative effect estimates.

**Table 1:** Summary of experimental parameters and outcomes reported in the included studies. Variables include Study, Virus strain and model, MOI or PFU/cell, Time point (hours post-infection, hpi), Species, Model system, Method, Outcome, Mean sequencing depth (million reads/sample), Differentially expressed genes (DEGs), and Observations. For the DEGs variable, individual gene symbols are listed when derived from RT-qPCR analyses, while total DEG counts are reported for RNA-seq results.

Study	ToxRTool Score	Virus Strain/Viral Model	MOI or PFU/Cell	RNA Collection Time (hpi)	Species	Model	Method	Outcome	Mean Sequencing Depth (Million Reads/Sample)	Results (DEGs)		Observations
										Upregulated	Downregulated	
Bulstrode et al., 2022 [34]	1	ZIKVPE243/mCherry	10	48–72 *	*H. sapiens*	Case: Primary HR GBM; Control: Primary MR GBM (in vitro).	RNA-seq	DEGs in highly refractory (HR) vs. moderately refractory (MR) GBM following ZIKV infection.	- **	13	6	* The study does not clearly state the post-infection time point used for the RNA-seq analysis. ** The study did not report sequencing metrics.
						Case: ZIKV-infected primary GBM; Control: Uninfected primary GBM (in vitro).		DEGs in ZIKV-infected vs. non-infected GBM.	- **	20	1	
Chen et al., 2018 [35]	2	ZIKVFSS13025/LAV	1	48	*H. sapiens*	Case: ZIKV-infected 4121 GSCs; Control: Uninfected 4121 GSCs (in vitro). *	RNA-seq	DEGs in ZIKV-infected vs. non-infected GBM.	38.2	528	19	* The study employed in vivo models; however, it did not conduct transcriptomic analyses on in vivo-derived cells.
						Case: ZIKV-infected 4121 GSCs; Control: Uninfected 4121 GSCs (in vitro). *	RT-qPCR	DEGs in ZIKV-infected vs. non-infected GBM.	-	*CDKN2B, CXCL10*, *CCL5*, *DDIT3*, *GADD45B*	*CCL2*	
Chen et al., 2022 [36]	3	ZIKVGZ01/WT	- *	24	*H. sapiens*	Case: ZIKV-infected primary (GBM#27) cells; Control: Uninfected primary (GBM#27) cells (in vitro).	RT-qPCR	DEGs in ZIKV-infected vs. non-infected GBM.	-	*IFNB1*, *CCL5*, *CXCL10*, *CXCL11*	-	* MOI not reported for these experimental conditions.
				48		Case: ZIKV-infected primary (GBM#27) cells; Control: Uninfected primary (GBM#27) cells (in vitro).		DEGs in ZIKV-infected vs. non-infected GBM.	-	*IFNB1*, *CCL5*, *CXCL10*, *CXCL11*	-	
				24		Case: ZIKV-infected primary (456 GSC) cells; Control: Uninfected primary (456 GSC) cells (in vitro).		DEGs in ZIKV-infected vs. non-infected GBM.	-	*CCL5*, *CXCL10*, *CXCL11*	-	
Chen et al., 2022 [36]	3	ZIKVGZ01/WT	- *	48	*H. sapiens*	Case: ZIKV-infected primary (456 GSC) cells; Control: Uninfected primary (456 GSC) cells (in vitro).	RT-qPCR	DEGs in ZIKV-infected vs. non-infected GBM.	-	*IFNB1*, *CCL5*, *CXCL10*, *CXCL11*, *RSAD2*, *IFIT1*, *IFIT2*, *IFIT3*	-	* MOI not reported for these experimental conditions.
				48		Case: ZIKV-infected primary (456 GSC) cells; Control: Anti-IFNAR1 + ZIKV-infected primary (456 GSC) cells (in vitro).		DEGs in ZIKV-infected vs. Anti-IFNAR1 + ZIKV-infected GBM.	-	*RSAD2*, *IFIT1*, *IFIT2*, *IFIT3*, *CXCL10*	-	
				24	*M. musculus*	Case: ZIKV-infected CT-2A cells; Control: Uninfected CT-2A cells (in vitro).		DEGs in ZIKV-infected vs. non-infected GBM.	-	*IFNA1*, *IFNB1*, *CCL5*, *CXCL9*, *CXCL10*, *CXCL11*	-	
				48		Case: ZIKV-infected CT-2A cells; Control: Uninfected CT-2A cells (in vitro).		DEGs in ZIKV-infected vs. non-infected GBM.	-	*IFNA1*, *IFNB1*, *RSAD2*, *IFIT1*, *IFIT2*, *IFIT3*, *IRF7*, *CCL5*, *CXCL9*, *CXCL10*, *CXCL11*	-	
				48		Case: ZIKV-infected CT-2A cells; Control: Anti-IFNAR1 + ZIKV-infected CT-2A cells (in vitro).		DEGs in ZIKV-infected vs. Anti-IFNAR1 + ZIKV-infected GBM.	-	*RSAD2*, *IFIT1*, *IFIT2*, *IFIT3*, *IRF7*	-	
				24		Case: ZIKV-infected GL261 cells; Control: Uninfected GL261 cells (in vitro).		DEGs in ZIKV-infected vs. non-infected GBM.	-	*CCL5*, *CXCL9*, *CXCL10*, *CXCL11*, *RSAD2*, *IFIT1*, *IFIT2*, *IFIT3*, *IRF7*	-	
Chen et al., 2022 [36]	3	ZIKVGZ01/WT	- *	48	*M. musculus*	Case: ZIKV-infected GL261 cells; Control: Uninfected GL261 cells (in vitro).	RT-qPCR	DEGs in ZIKV-infected vs. non-infected GBM.	-	*CCL5*, *CXCL9*, *CXCL10*	-	* MOI not reported for these experimental conditions. ** The study does not clearly specify the viral strain used in the experiment. *** The list of DEGs from this experiment was not made available.
				24		Case: ZIKV-infected GL261 cells; Control: Anti-IFNAR1 + ZIKV-infected GL261 cells (in vitro).		DEGs in ZIKV-infected vs. Anti-IFNAR1 + ZIKV-infected GBM.	-	*RSAD2*, *IFIT1*, *IFIT2*, *IFIT3*, *IRF7*	-	
		ZIKVFSS13025/WT	0.2	288		Case: Anti-PD-L1 + ZIKV-infected C57BL/6N mice inoculated with CT-2A; Control: ZIKV-infected C57BL/6N mice inoculated with CT-2A (in vivo).		DEGs in Anti-PD-L1 + ZIKV-infected vs. ZIKV-infected GBM.	-	*CCL5*, *CXCL10*, *CXCL11*, *ISG15*, *RSAD2*, *IFIT1*, *IL6*, *IFNB1*, *IFNG*	-	
				720		Case: Anti-PD-L1 + ZIKV-infected C57BL/6N mice inoculated with CT-2A; Control: ZIKV-infected C57BL/6N mice inoculated with CT-2A (in vivo).		DEGs in Anti-PD-L1 + ZIKV-infected vs. ZIKV-infected GBM.	-	*IL6*, *IFNB1*	-	
				360		Case: ZIKV-infected C57BL/6N mice inoculated with GL261; Control: Uninfected C57BL/6N mice inoculated with GL261 (in vivo).	RNA-seq	DEGs in ZIKV-infected vs. non-infected GBM.	44.3	- ***	- ***	
		ZIKVFSS13025 or ZIKVGZ01/WT **		528		Case: Anti-PD-L1 + ZIKV-infected C57BL/6N mice inoculated with GL261; Control: ZIKV-infected C57BL/6N mice inoculated with GL261 (in vivo).		DEGs in Anti-PD-L1 + ZIKV-infected vs. ZIKV-infected GBM.	43.2	- ***	- ***	
Chen et al., 2023 [37]	1	ZIKVGZ01/WT	1	24	*H. sapiens*	Case: ZIKV-infected U251 cells; Control: Uninfected U251 cells (in vitro).	RT-qPCR	DEGs in ZIKV-infected vs. non-infected GBM.	-	*TNF*	-	-
				48				DEGs in ZIKV-infected vs. non-infected GBM.	-	*OASL*, *OAS2*, *IFIT2*, *IFIT3*, *ISG15*, *TRIM22*, *TNF*	-	
				96				DEGs in ZIKV-infected vs. non-infected GBM.	-	*TNF*	-	
				144				DEGs in ZIKV-infected vs. non-infected GBM.	-	*TNF*	-	
				48			RNA-seq	DEGs in ZIKV-infected vs. non-infected GBM.	21.8	3460	1505	
Zhu et al., 2017 [9]	1	ZIKVParaiba_01/2015 and ZIKVDakar41519/WT	5	36–48 *	*H. sapiens*	Case: ZIKV-infected 3565 and 4121 GBM cells; Control: ZIKV-infected 3565 and 4121 NSTC cells (in vitro). **	RT-qPCR	DEGs in ZIKV-infected GBM vs. ZIKV-infected NSTC.	-	-	*IFNAR1*, *STAT1*, *IRF1*, *IFIT1*, *OAS2*, *IFH1*	* The study does not clearly state the post-infection time point used for the RNA-seq analysis. Also, the study used two ** and three *** different GBM cell lines for the transcriptomic analysis.
						Case: ZIKV-infected 3565, 387, and 4121 cells; Control: Uninfected 3565, 387, and 4121 cells (in vitro). ***	RNA-seq	DEGs in ZIKV-infected vs. non-infected GBM.	62.7	1338	726	

## 3. Results

### 3.1. Study Selection

The literature search retrieved 136 records from the two databases. The cross-referencing screening on the retrieved reviews added 3 other primary articles, leading to 139 records in total. We excluded 65 studies on the title and abstract screening step. Another 69 records were excluded after full-text assessment: 43 investigated ZIKV infection in GBM models without performing differential expression analysis; 18 were unavailable in full text (10 abstracts, 6 commentaries, and 1 project); 3 were reviews; 2 were non-GBM studies; 2 were preprints; 1 was a non-ZIKV study; and 1 was a restricted-access article (Table S2). Ultimately, five studies met the eligibility criteria and were included in the review (Figure S1) [9,34,35,36,37].

### 3.2. Study Characteristics

Data extracted from the methods, results, and figures sections of the eligible studies were combined to construct the outcome table (Table 1).

The included studies employed a variety of ZIKV strains and experimental models. Bulstrode et al. (2022) [34] and Chen et al. (2018) [35] used a recombinant reporter ZIKV-mCherry construct and an attenuated virus, respectively. Chen et al. (2022) [36], Chen et al. (2023) [37], and Zhu et al. (2017) [9] utilized wild-type (WT) ZIKV strains. Across the five studies, five distinct strains were reported as follows: ZIKVPE243 and ZIKVParaiba_01/2015 (American), ZIKVFSS13025 and ZIKVGZ01 (Asian), and ZIKVDakar41519 (African) with some overlap between studies. Although certain studies used multiple strains, transcriptomic analyses were typically conducted using only one. Notably, Chen et al. (2022) [36] was the only study to report DEGs derived from two distinct strains (ZIKVFSS13025 and ZIKVGZ01).

Diversity was also observed in the cellular and murine models employed to evaluate the virus infection. Four studies employed primary GBM cultures derived from patient tumors [9,34,35,36], while two studies used immortalized cell lines—U251 (human, Sigma-Aldrich #9063001, St. Louis, MO, USA), CT-2A (murine, Sigma-Aldrich #SCC194), and GL261 (murine, BCRJ #0299) [36,37]. In vivo models were based on the C57BL/6 mouse strain, used by three studies [9,35,36]; in addition, Chen et al. (2018) [35] also employed BALB/c nude mice to evaluate ZIKV-mediated tumor inhibition. Importantly, only Chen et al. (2022) [36] performed transcriptomic profiling on in vivo-derived GBM tissue, making it the sole source of in vivo DEG data included in the outcome table (Table 1).

Regarding the experimental design, the included studies employed a range of multiplicities of infection (MOIs), from 0.2 to 10 (median = 1; SD = 4.12). Post-infection RNA collection time also varied considerably between studies that worked with in vitro models, ranging from 24 to 144 h (median = 48; SD = 30.63). One study [36] did not clearly report the MOI, and two studies [9,34] lacked information about RNA collection time in their methods. In both cases, the corresponding data were not included in the outcome table and statistical analysis. The studies presented diverse outcomes assessed primarily by RT-qPCR (16 comparisons; 72.72%) and, to a lesser extent, by RNA-seq (6 comparisons; 27.27%). All five studies investigated DEGs between ZIKV-infected and non-infected GBM samples. Furthermore, some studies explored case/control comparisons within the infected groups, aiming to investigate the following: the effect of antibody neutralization on gene expression during infection [36], transcriptomic differences between GBM lines classified as highly and moderately refractory to ZIKV infection [34], and the expression pattern of GBM vs. healthy nuclear transfer stem cells (NTSCs), both infected with ZIKV [9].

A ToxRTool [24] reliability score was assigned to all five studies, as they all included in vitro experiments. Three studies were classified as “reliable without restrictions” (score 1) [9,34,37], one as “reliable with restrictions” (score 2) [35], and one as “not reliable” (score 3) [36]. The three studies that conducted in vivo experiments were further evaluated with SYRCLE’s Risk of Bias Tool, which does not provide an overall quality score. In general, these studies received a “yes” judgment, indicating a low risk of bias, for items 2, 9, and 10, which assess the description of baseline characteristics, selective outcome reporting, and other potential sources of bias, respectively. Conversely, most studies received a “no” judgment, indicating a high risk of bias, for items 1 and 3–8 (Table 2).

### 3.3. Twelve Genes Were Consistently Upregulated in ZIKV-Infected GBM Cell Lines

**Figure 1:**
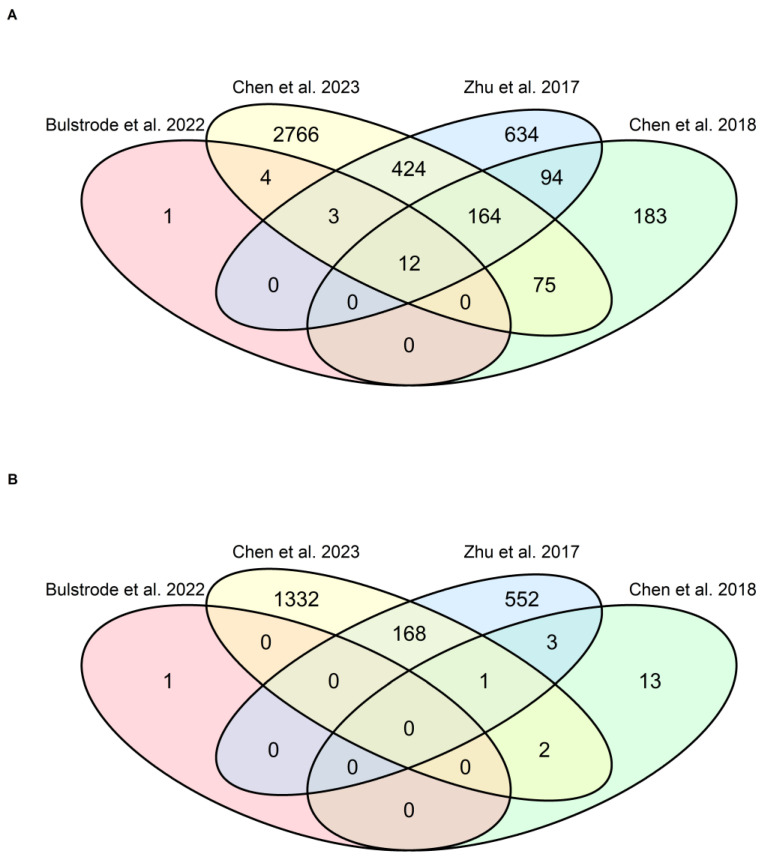
Differentially expressed genes (DEGs) in ZIKV-infected human GBM cells [9,34,35,37]. Venn diagrams displaying the number of upregulated (**A**) and downregulated (**B**) genes identified by RNA sequencing in ZIKV-infected (case) vs. non-infected (control) human GBM cells across four of the five studies included in this review. *DNAJB9*, *SESN2*, *PMAIP1*, *PPP1R15A*, *KLF4*, *ATF3*, *IFNB1*, *IFNL1*, *ANKRD33B*, *ZC3HAV1*, *OASL*, and *CCL5* were consistently upregulated across all studies. In total, 145 genes showed inconsistent regulation directions across different studies.

For the sake of comparability among studies, only DEGs derived from RNA sequencing experiments comparing the transcriptomic profiles of ZIKV-infected vs. non-infected human GBM cells were collected for further analysis (Table S1). Chen et. al., 2022 [36], which assessed the transcriptome of ZIKV-infected murine GBM, was therefore not included in the dataset. Venn diagrams (Figure 1) presenting the intersection of the absolute number of upregulated (A) and downregulated (B) genes were constructed.

Overall, a total of 4360 genes were reported as upregulated and 2072 as downregulated across the four studies [9,34,35,37] included in this analysis (Figure 1). In each of them, the number of upregulated genes exceeded the number of downregulated genes. Notably, 145 genes (2.3%) were found to be upregulated in at least one study and downregulated in at least another study, suggesting inconsistent regulation patterns across different experimental models. Twelve genes were reported as upregulated in the four studies: DnaJ homolog, subfamily B, member 9 (*DNAJB9*; endoplasmic reticulum chaperone), Sestrin-2 (*SESN2*; oxidative stress sensor), Phorbol-12-Myristate-13-Acetate-Induced Protein 1 (*PMAIP1*; pro-apoptotic factor), Protein phosphatase 1 regulatory subunit 15A (*PPP1R15A*; stress-induced regulator of protein synthesis), KLF Transcription Factor 4 (*KLF4*; cell cycle and differentiation regulator), Activating transcription factor 3 (*ATF3*; immediate early stress response gene), Interferon Beta 1 (*IFNB1*; type I interferon), Interferon-lambda 1 (*IFNL1*; type III interferon), Ankyrin Repeat Domain 33B (*ANKRD33B*; transcriptional regulator with unclear function), Zinc Finger CCCH-Type Antiviral Protein 1 (*ZC3HAV1*; antiviral RNA-binding protein), 2′-5′-Oligoadenylate Synthetase Like (*OASL*; interferon-stimulated antiviral enzyme), and CC Motif Chemokine Ligand 5 (*CCL5*; proinflammatory chemokine). Most of these genes point to a coordinated innate defense response to arboviruses (Figure 1A). In contrast, no gene was found to be downregulated across all four studies (Figure 1B).

### 3.4. Pathway Analysis Identified 23 Enriched Molecular Processes

**Figure 2:**
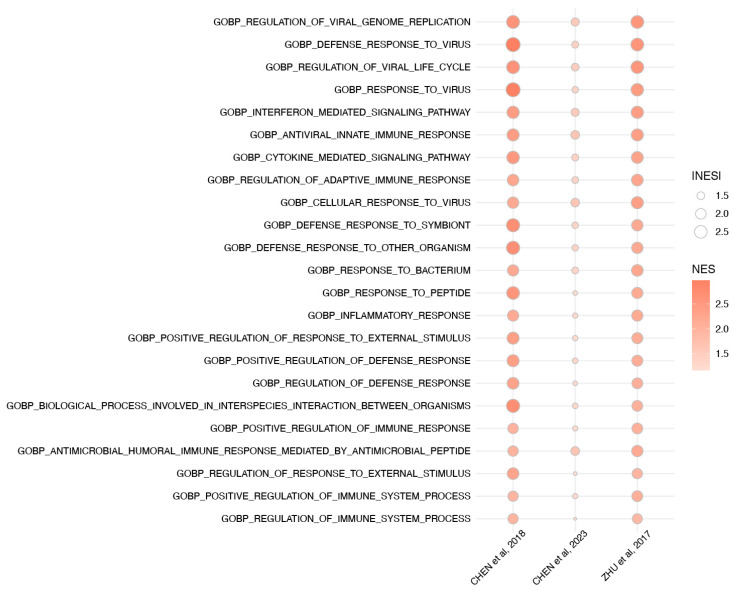
Concordant enrichment of Gene Ontology Biological Processes (GO:BP) across three ZIKV-infected GBM datasets [9,35,37]. Dot plot representing significantly enriched GO:BP pathways (adjusted *p* < 0.01, |NES| > 1) found in the intersection of three transcriptome differential expression analyses. Pathways are ordered by normalized enrichment score (NES). Each dot represents the NES of the corresponding pathway in each dataset, where color indicates the NES direction and intensity, and size reflects the adjusted *p* value.

A pathway analysis was performed for the three studies that provided the complete list of DEGs or the count results in ZIKV-infected GBM, either within the main article or through publicly available databases: Chen et al. 2018 [35] (mean depth = 38.2 M reads/sample), Chen et al. 2023 [37] (mean depth = 21.8M reads/sample), and Zhu et al. 2017 [9] (mean depth = 62.7 M reads/sample) (Table 1). Differential expression results from Zhu 2017 [9] (GSE102924) and Chen 201833 (GSE114907) were imported directly from processed tables. For Chen 2023 [37] (GSE234128), raw count data were extracted for the U251 cell line under ZIKV-infected and non-infected (NF) conditions. To identify conserved biological programs across distinct glioma-related conditions, we performed gene set enrichment analysis (GSEA) using the GO:BP collection on three transcriptomic comparisons: (i) Zhu et al., 2017 [9], glioma stem cells infected with ZIKA virus Dakar strain, (ii) Chen et al. 2018 [35], Zika virus-infected glioblastoma neurospheres; and (iii) Chen et al. 2023 [37], U251 glioma cells infected with Zika virus. Differential expression analyses were conducted using *DESeq2* [30] for count-based datasets and retrieved directly when available. Pathways present in all the studies with a *p* adjusted lower than 0.01 were included in the analysis (Figure 2).

Full pathway results are available in Table S3. Chen et al., 2018 [35] yielded 384 enriched pathways, Chen et al., 2023 [37] yielded 455 enriched pathways, and Zhu et al., 2017 [9] yielded 763 enriched pathways.

The three studies [9,35,37] shared 75 enriched pathways; from these, we display the top 23 NES pathways (Figure 2). These included the regulation of viral genome replication and viral cycle, which in turn activated cellular pathways such as the cellular response to viruses, defense response to viruses, regulation of immune system process, positive regulation of immune response, cytokine-mediated signaling, antiviral innate immune response, and interferon-mediated signaling. These pathways collectively reflect the activation of innate immune programs, particularly those centered on type I and type III interferon signaling against virus replication in a cellular scenario. Together, these findings reiterate the strong concordance across datasets; common immune and antiviral programs are consistently enriched; while dataset-specific pathways exhibited lower enrichment and adjusted *p* values within individual studies, indicating context-dependent magnitudes of pathway activation.

## 4. Discussion

A search conducted up to 5 March 2025 identified 47 studies investigating ZIKV infection in GBM models. Of these, 43 explored various strategies to test their hypotheses, either through targeted approaches or by directly assessing the effects of ZIKV on GBM models. Only five studies examined the transcriptomic profiles of ZIKV-infected GBM cells, and none addressed epitranscriptomic signatures. Accordingly, while the transcriptional patterns discussed here were reproducible across the available datasets, the limited number of studies necessarily constrains the scope and strength of the conclusions that can be drawn at this stage. The scarcity of differential expression data highlights the need to expand these studies, with particular attention to maintaining standardized experimental designs to enable comparability.

This limitation was evident in the present review, as the five included studies varied substantially in experimental design, including viral strains, in vitro and in vivo models, infection time points and doses (MOI), research objectives, and methodologies. While such heterogeneity serves an exploratory function in the early investigation of a novel phenomenon, it also presents a major barrier to data uniformity and cross-study comparability. Differences in ZIKV strains, infection time points, and experimental models (cell lines vs. animal models) inherently influence transcriptional responses, thereby limiting the consistency of observed gene expression patterns across studies [38].

In addition, the predominance of in vitro systems constrains the direct extrapolation of these transcriptomic signatures to human GBM tumors. Although cell-based models are indispensable for mechanistic studies and offer high experimental control, they fail to recapitulate key features of the tumor microenvironment, such as immune infiltration, stromal interactions, vascularization, and three-dimensional architecture, that can critically modulate viral tropism and host transcriptional responses in vivo [39]. The fact that only a single study [36] reported transcriptomic data derived from in vivo GBM tissue further limits biological generalizability and underscores the need for additional in vivo translational investigations to validate whether these gene expression patterns are maintained in intact tumors. Regarding strain variability, although American ZIKV lineages have been associated with more pronounced neurological manifestations compared to Asian and African isolates [40,41], this association was not reflected in a higher number of upregulated genes, an expected consequence of stronger transcriptional responses, in GBM models infected with these strains [9,34].

The discrepancy in the number of upregulated genes among studies is more likely influenced by under-reporting than by strain-specific characteristics: the complete list of DEGs and sequencing quality metrics were not available in Bulstrode et al. (2022) [34], precluding a definitive analysis. Comprehensive reporting of such information, particularly mean RNA sequencing depth, is crucial, as this metric is positively correlated to the likelihood of identifying rare transcripts. For this reason, a minimum mean depth of 25M reads/sample is recommended for transcriptomic studies [42]. Among the five studies reviewed here, two met this requirement [9,35], one reached 21.8 M reads/sample, slightly below the threshold [37], and two did not report sequencing metrics [34,36]. The optimization of experimental models has long been recognized as a major challenge in studies conducting transcriptomic analyses, as it critically affects data reproducibility and biological interpretation [43,44], reinforcing the urgent need for consistency in protocols.

The lack of essential methodological information due to poor reporting was not limited to sequencing metrics and constituted a barrier to data synthesis. This reporting bias was evaluated using quality checklists. Although some studies failed to report basic protocol elements, such as the use of negative controls, number of replicates [35], cell line origin, MOI, and route of administration [36], we included all studies in our analysis regardless of their ToxRTool [26] score, as our adapted version of the tool has not yet been validated. Because the synthesis was descriptive and not based on pooled quantitative estimates, excluding studies based on ToxRTool scores would not meaningfully alter the qualitative patterns identified across datasets. Nevertheless, the classification of Chen et al., 2022 [36] as “not reliable” should be taken into account when interpreting that study’s results and conclusions. For studies employing in vivo models, evaluation with the SYRCLE RoB tool revealed a high risk of bias for most checklist items. With the exception of items 2 (baseline characteristics), 9 (selective outcome reporting), and 10 (other potential sources of bias), studies did not meet the requirements for proper allocation and blinding across several experimental steps. Despite this, the checklist does not recommend assigning a summary score, as this “… inevitably involves assigning ‘weights’ to specific domains in the tool, and it is difficult to justify the weights assigned,” which also “… might differ per outcome and per review” [28]. Therefore, we did not weigh the studies’ results based on their SYRCLE evaluation. However, this choice should be acknowledged as a potential source of heterogeneity and bias that may weaken the strength and generalizability of our conclusions, emphasizing the need for greater standardization and transparency in future ZIKV-GBM transcriptomic studies.

In total, twelve genes were found to be consistently upregulated across all included studies: *DNAJB9*, *SESN2*, *PMAIP1*, *PPP1R15A*, *KLF4*, *ATF3*, *IFNB1*, *IFNL1*, *ANKRD33B*, *ZC3HAV1*, *OASL*, and *CCL5*. This gene set integrates the stress response regulators *DNAJB9* [45], *SESN2* [46], *PPP1R15A* [47], *ATF3* [48], and *KLF4* [49], with the canonical antiviral/innate immune mediators *IFNB1* [50], *IFNL1* [51], *ZC3HAV1* [52], *OASL* [53], and *CCL5* [54]. Their coordinated induction suggests that ZIKV infection elicits a multifaceted response in GBM cells, where cellular stress pathways converge with type I/III interferon signaling and chemokine-mediated immune activation. Importantly, the recurrence of this specific transcriptional signature across independent studies indicates that these genes likely represent conserved components of the cellular response to ZIKV infection in GBM, rather than context-dependent or model-specific effects. Mechanistically, this implies that ZIKV triggers endoplasmic reticulum stress and unfolded protein response elements, which intersect with interferon-stimulated gene networks to promote immune recruitment. Such convergence between stress-adaptive pathways and antiviral signaling is a common feature of productive viral infections and reflects the tight coupling between viral sensing, cellular homeostasis, and innate immune activation. This coordinated innate immune response in GBM cells may underlie the antiviral resistance mechanisms observed during ZIKV infection. Beyond direct cytopathic effects, immune system engagement is an essential component of oncolytic virus activity. The induction of ISGs contributes to the inflammation of the tumor microenvironment, promoting immune cell recruitment and antigen presentation rather than solely reflecting antiviral restriction [6]. In this context, the recurrent upregulation of innate immune mediators observed across studies may indirectly elicit antitumor responses without implying direct evidence of tumor cell elimination. The limited gene overlap reflects the fact that GBM comprises multiple molecular subtypes with distinct baseline transcriptomic profiles, which may modulate the host response to infection. Thus, the small set of common DEGs may represent the core, most conserved elements of ZIKV-induced stress and immune activation in glioblastoma models [55], providing mechanistic insight into how GBM cells integrate viral sensing with downstream transcriptional programs.

Consistent with our findings, transcriptomic analyses of other oncolytic viruses in GBM models have revealed that host gene expression changes are dominated by immune- and infection-related responses rather than virus-specific cytopathic signatures. For example, ex vivo infection of patient-derived GBM cells with herpes simplex virus type 1 (HSV1) was characterized by the upregulation of genes encoding immunogenic and secreted proteins linked to immune activation [56]. Together with our results, these observations support the notion that oncolytic activity in GBM is largely associated with conserved, host-driven antiviral and immune transcriptional programs that contribute to immunomodulation rather than virus-specific effects.

Interestingly, among the consistently upregulated genes, *PMAIP1* (*NOXA*) stands out as the only canonical pro-apoptotic effector, in contrast to the other genes, which are primarily associated with cellular stress responses and antiviral immune signaling, being commonly observed in a wide range of viral infections. The induction of *PMAIP1* is particularly relevant, as it encodes a BH3-only protein that directly promotes mitochondrial outer membrane permeabilization and caspase activation, thereby committing the cell to apoptosis [57]. Notably, *PMAIP1* induction appears to be selective, as other BH3-only proteins such as *BIM* or *PUMA* were not consistently identified across studies. This specificity suggests that ZIKV infection engages a distinct apoptotic program, potentially linked to interferon signaling or virus-induced cellular stress rather than to generalized apoptotic cues such as growth factor withdrawal or DNA damage. Indeed, *PMAIP1* has been reported to be inducible downstream of type I interferon signaling and integrated stress responses, positioning it at the interface between antiviral defense and mitochondrial apoptosis. Within this context, *PMAIP1* upregulation may reflect a regulated cellular decision to eliminate infected cells under conditions of sustained antiviral and stress signaling, rather than an incidental consequence of cytotoxicity. This selective activation suggests that ZIKV infection may not only trigger a generic antiviral response but also engage GBM cell death pathways, positioning *PMAIP1* as a promising target for further functional studies aimed at dissecting virus–host interactions.

The presence of *ANKRD33B* (a transcript expressed mainly in brain and kidney tissue) [58] among the upregulated genes is also notable. Although its functional role in this context remains unclear due to the limited research available, the conserved upregulation observed across studies highlights it as worthy of further investigation. *ANKRD33B* contains ankyrin repeat domains, which are commonly involved in protein–protein interactions and the assembly of signaling complexes. This structural feature raises the possibility that *ANKRD33B* may participate in the modulation of host signaling pathways activated during ZIKV infection, potentially acting as a scaffolding or regulatory factor. While speculative, the repeated identification of *ANKRD33B* across heterogeneous datasets suggests that it may represent an underexplored component of the GBM cellular response to ZIKV, underscoring the need for targeted functional studies to clarify its role in viral infection biology. No gene was found to be consistently downregulated across the four studies. Moreover, the number of downregulated genes in each study was substantially lower than that of upregulated genes. This pattern may reflect ZIKV’s relatively mild clinical manifestations, potentially indicating a lower degree of host gene suppression by its replication machinery compared to other medically relevant arboviruses [59,60]. The pathway analysis extended the findings from the overlap investigation of commonly upregulated genes. The 23 commonly enriched pathways point toward core mechanisms of the antiviral innate immune response, which operate in a coordinated manner to detect, contain, and eliminate viral infections by mediating viral RNA degradation and inhibiting viral replication, while simultaneously regulating the intensity and duration of the immune response.

### Review Limitations

Important limitations of this work must be acknowledged: restriction to open-access studies, the use of only two bibliographic databases (PubMed and Google Scholar), which may have resulted in the omission of relevant studies indexed elsewhere, limiting inclusion for pathway analysis to studies reporting complete DEG lists, a criterion that, while adopted to ensure transparency and reproducibility, may have led to the exclusion of otherwise high-quality studies and biased the synthesis toward datasets with more comprehensive reporting practices, and reliance on published transcriptomic datasets, which may have varying reporting standards, constituted limitations to the review process. Additionally, because only a subset of the included studies obtained DEG lists via RNA-seq, pathway-level integration was necessarily restricted, which may have limited the detection of consistently enriched biological processes across heterogeneous experimental designs. Also, the small number of eligible studies, the heterogeneity of experimental designs, and inconsistencies in methodological reporting introduce potential biases that constrain the strength of our conclusions. Moreover, the adapted ToxRTool [26] instrument used in our quality assessment has not yet been formally validated. These limitations underscore the need to replicate transcriptomic profiling of this virus–cell interaction under uniformed protocols with rigorous data reporting, as well as to develop appropriate quality assessment instruments designed specifically for the context of infected in vitro models.

## 5. Conclusions

This systematic review highlights the convergent, although limited, transcriptomic evidence available on ZIKV infection in GBM models, revealing a consistent upregulation of key interferon-related genes and pathways involved in the antiviral innate immune response. Notably, the pro-apoptotic effector *PMAIP1* and the poorly characterized gene *ANKRD33B* emerge as potentially relevant targets for future investigations of ZIKV-induced apoptosis in GBM cells. Integrative approaches that incorporate the epitranscriptome of ZIKV-infected GBM cells as an additional omics layer are warranted to further elucidate the molecular interplay of ZIKV-GBM interactions. Beyond transcriptomics, future studies should aim to integrate epigenetic, proteomic, and metabolomic data to enable a more comprehensive multi-omics characterization of the molecular interplay between ZIKV infection and GBM cellular responses. Future systematic reviews should explore complementary experimental strategies, such as immunological blockade, gene knockout, and viability assays, in order to elucidate the mechanisms underlying ZIKV’s viral cycle in GBM and to promote greater standardization of study designs in the research field.

## Figures and Tables

**Table 2:** SYRCLE’s Risk of Bias (RoB) tool evaluation matrix. Summary of the categorical assessment for each item pointed by the instrument. 1—Sequence generation, 2—baseline characteristics, 3—allocation concealment, 4—random housing, 5—experimental blinding, 6—random outcome assessment, 7—outcome assessment blinding, 8—incomplete outcome data, 9—selective outcome reporting, 10—other sources of bias. Responses are color-coded as follows: Yes (green) and No (red).

Author	SYRCLE Question									
	1	2	3	4	5	6	7	8	9	10
Chen et al., 2018 [35]	**-**	+	-	-	-	-	-	-	+	+
Chen et al., 2022 [36]	**-**	+	-	-	-	+	-	-	+	+
Zhu et al., 2017 [9]	**-**	+	-	-	-	-	-	-	+	-
Yes								**+**		
No								**-**		

## Data Availability

The datasets generated during the current study are available from the corresponding author upon reasonable request. Expression profile data analyzed in this study were obtained either from the figures and full-text reporting or from the Gene Expression Omnibus (GEO) at GSE102924, GSE114907, and GSE234128. The processed differential expression tables and all the codes used for the analyses are available at https://github.com/iza-mcac/2025-09-Systematic-Zikv-Glio (26 January 2026).

## Supplementary Materials

The following supporting information can be downloaded at: https://www.mdpi.com/article/10.3390/v18020249/s1, Supplementary File S1: list of terms used in each database during the searching step of the PRISMA flowchart; Figure S1: PRISMA flowchart; Table S1: full list of DEGs included in the overlapping (Venn diagrams) analysis; Supplementary File S2: adapted ToxRTool for the evaluation of oncolytic virus studies; Table S2: list of studies excluded after full-text assessment, with reasons. Table S3: full pathway analysis results.

## Abbreviations

The following abbreviations are used in this manuscript:

GBM	Glioblastoma
ZIKV	Zika virus
DEGs	Differentially expressed genes
WHO	World Health Organization
CNS	Central nervous system
OVs	Oncolytic viruses
RNA-seq	RNA sequencing
PROSPERO	International Prospective Register of Systematic Reviews
PRISMA	Preferred Reporting Items for Systematic reviews and Meta-analysis
MOI	Multiplicity of infection
RT-qPCR	Polymerase chain reaction
HGNC	HUGO Gene Nomenclature Committee
DE	Differential expression
GO:BP	Gene Ontology Biological Process
NES	Normalized enrichment score
HR	Highly refractory
MR	Moderately refractory
WT	Wild-type
NTSCs	Nuclear transfer stem cells
RoB	Risk of bias
NF	Non-infected
GSEA	Gene set enrichment analysis
PAMPs	Pathogen-associated molecular patterns
DAMPs	Damage-associated molecular patterns
HSV1	Herpes simplex virus type 1
