# Supplementary material for: Transcriptomic Profile of Glioblastoma Cells Infected with Zika Virus: A Systematic Review and Pathway Analysis
Source: Viruses. 2026 Feb 15;18(2):249. doi: 10.3390/v18020249 (PMC12945271; doi:10.3390/v18020249)
Supplement: Supplementary file 1 [file viruses-18-00249-s001.zip › Suplementary file S1.docx]

**PubMed:**

(ZIKV[Title/Abstract] OR Zika[Title/Abstract] OR zika[Title/Abstract] OR ZIKA[Title/Abstract] OR Zikavirus[Title/Abstract] OR ZikaVirus[Title/Abstract] OR zikaVirus[Title/Abstract] OR ZIKAVIRUS[Title/Abstract]) AND (Glioblastoma[Title/Abstract] OR GLIOBLASTOMA[Title/Abstract] OR glioblastoma[Title/Abstract] OR gbm[Title/Abstract] OR GBM[Title/Abstract] OR Gbm[Title/Abstract] OR GBCs[Title/Abstract] OR gbcs[Title/Abstract] OR Gbcs[Title/Abstract] OR glioma[Title/Abstract] OR GLIOMA[Title/Abstract] OR Glioma[Title/Abstract])

**Google Scholar:**

(intitle:ZIKV OR intitle:Zika OR intitle:Zikavirus) AND (intitle:Glioblastoma OR intitle:GBM OR intitle:glioma OR intitle:GBCs)
