# Supplementary figures and images for: Transcriptomic Profile of Glioblastoma Cells Infected with Zika Virus: A Systematic Review and Pathway Analysis
Source: Viruses. 2026 Feb 15;18(2):249. doi: 10.3390/v18020249 (PMC12945271; doi:10.3390/v18020249)

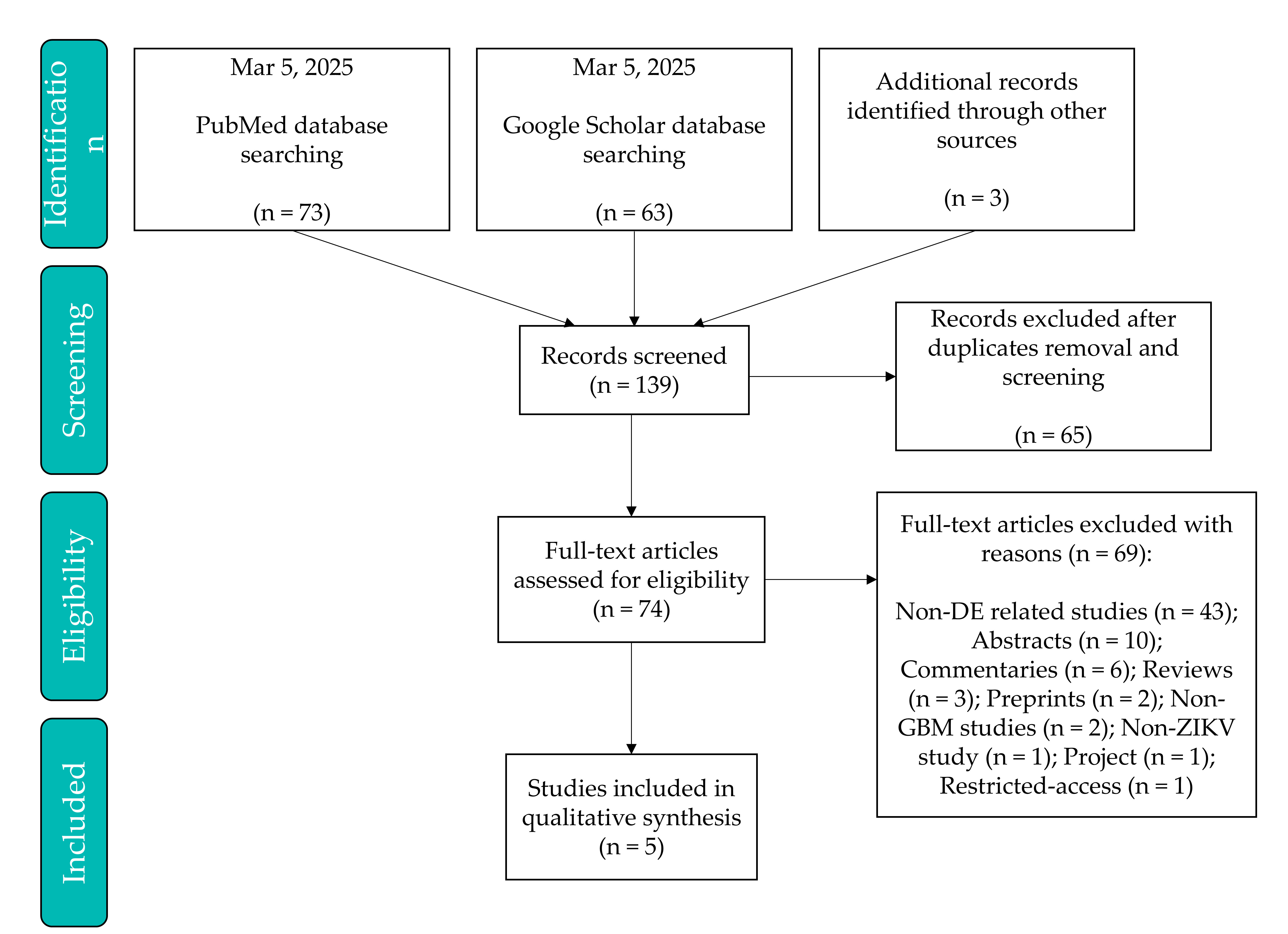

Supplement: Supplementary file 1 [file viruses-18-00249-s001.zip › FigS1.PNG]
